# The Role of Ferroptosis in Atrial Fibrillation: A Promising Future

**DOI:** 10.31083/j.rcm2504127

**Published:** 2024-04-01

**Authors:** Jia-Bin Zhou, Ling-Ling Qian, Dan Wu, Ru-Xing Wang

**Affiliations:** ^1^Department of Cardiology, The Affiliated Wuxi People's Hospital of Nanjing Medical University, Wuxi People's Hospital, Wuxi Medical Center, Nanjing Medical University, 214023 Wuxi, Jiangsu, China

**Keywords:** atrial fibrillation, ferroptosis, mechanism, therapeutic implication

## Abstract

Atrial fibrillation (AF) is one of the most common cardiac arrhythmias, with its diagnosis being closely tied to higher rates of cardiovascular morbidity and mortality. AF is associated with a range of dangerous complications including stroke and heart failure, making it a key driver of healthcare spending and a major threat to global public health. The precise mechanisms that govern AF incidence and the onset of related complications, however, remain uncertain. Ferroptotic cell death has been the focus of rising interest in the cardiac arrhythmias, and there is recent evidence supporting a role for atrial ferroptosis as a mediator of AF development. Interventional strategies focused on ferroptotic activity, such as novel ferroptosis inhibitors, have also shown promise as a means of protecting against AF through their ability to reduce iron overload. In this review, we provide a summary of the proposed mechanisms whereby ferroptosis contributes to the pathophysiology of AF and their therapeutic implications.

## 1. Introduction

Atrial fibrillation (AF) is a commonly diagnosed form of cardiac arrhythmias which typically results from rapid, erratic atrial signals and is classified as paroxysmal, persistent, long-running persistent, or permanent AF [1]. Approximately 33 million individuals throughout the globe are impacted by AF, and this number is forecast to double over the next four decades [2]. AF is typically related to a variety of other forms of cardiovascular diseases such as hypertension, cardiomyopathy, and coronary artery disease, potentially contributing to heart failure, stroke, thromboembolism, and potential death [3]. AF is a multifactorial and highly complex disease such that its precise pathological basis remains elusive, although structural and electrical remodeling, abnormal calcium homeostasis, and dysfunction of the autonomic nervous system are thought to contribute [4].

First proposed as a unique type of cell death in 2012 [5], ferroptosis is associated with regulatory proteins, biochemical characteristics, and morphological phenotypes which is distinct from apoptosis, autophagy, necroptosis, or pyroptosis. At a morphological level, cells undergoing ferroptotic death exhibit reductions in mitochondrial volume, increased membrane bilayer density, and the partial or total loss of cristae within the mitochondria. Reduced intracellular glutathione (GSH) expression, decreased glutathione peroxidase 4 (GPX4) activity, and subsequent accumulation of reactive oxygen species (ROS) and lipid peroxidation are biochemical features of ferroptosis [6]. At the genetic level, a number of iron homeostasis- and lipid peroxidation-related genes have been found to be associated with this process [7]. When iron overload occurs, excessively high levels of iron can be deposited within cells, contributing to irreversible tissue injury and associated organ failure [8].

Oxidative damage is the central pathogenesis of ferroptosis, arising primarily as a result of changes in mitochondrial homeostasis attributable to the accumulation of overly high levels of iron-dependent lipid peroxidation byproducts [9]. Physiological processes including those responsible for the metabolic processing of lipids, amino acids, and iron all shape the incidence of ferroptosis. There is increasingly strong evidence that ferroptotic activity is involved in a range of cardiovascular diseases, such as arrhythmias, cardiomyopathy, and myocardial infarction [10]. Myocardial hemosiderin deposition in patients with lethal epilepsy has recently been detected, further emphasizing the potentially central role that ferroptosis may play in the mechanistic processes underlying cardiac arrhythmias [11]. The activity of sodium, potassium, and calcium channels can be adversely impacted by iron overload, disrupting appropriate cardiac electrophysiology and codifying the biophysical association between iron ions and arrhythmias [12]. As the most commonly diagnosed form of cardiac arrhythmias, AF is also emerging as a particularly prominent focus in the field of ferroptosis [13].

## 2. General Mechanisms of AF

Atrial electrical remodeling is a crucial factor in developing the AF substrate [14]. This remodeling process predominantly involves reductions in atrial effective refractory period (ERP), atrial myocyte action potential duration (APD), and the emergence of conduction disturbances [15]. The APD and their repolarization are determined by a delicate balance between various inward and outward currents. These currents include L-type ${\mathrm{Ca}}^{2+}$ currents ($I_{\text{Ca,L}}$) and late ${\mathrm{Na}}^{+}$ currents ($I_{\text{Na,L}}$), along with the activity of several voltage-gated $K^{+}$ channels ($K_{\text{v}}$) [16].

AF and atrial fibrosis are closely interrelated, as AF exacerbates atrial fibrosis, leading to atrial remodeling. This remodeling creates substrates that further promote AF [17]. Myocardial fibrosis, a key indicator of atrial structural remodeling, disrupts myofibril continuity, impedes the formation of connexin-rich tight junctions, and compromises cardiac electrical conduction. This results in slowed conduction and conduction blocks [18]. Moreover, interactions between cardiomyocytes and fibroblasts/myofibroblasts can modify cardiomyocyte electrical properties, trigger ectopic discharges, and facilitate AF development [19].

The heart is richly supplied with autonomic nerves. Both the sympathetic and parasympathetic nervous systems contribute to AF development. Consequently, autonomic dysfunction is pivotal in the onset, progression, and intricacy of AF [20]. Evidence from a large cohort study indicates that cardiac autonomic dysfunction escalates AF incidence [21]. Additionally, AF can induce changes in autonomic tone and cardiac autonomic remodeling [22]. This indicates a bidirectional relationship between autonomic dysfunction and AF.

A prior study has reported that aberrant intracellular ${\mathrm{Ca}}^{2+}$ homeostasis serves as a significant aspect of the pathophysiology of AF, playing a crucial role in atrial remodeling [23]. Anomalies in the density of ${\mathrm{Ca}}^{2+}$ channels result in diminished atrial APD, inducing atrial electrical remodeling [24]. Concurrently, elevated intracellular ${\mathrm{Ca}}^{2+}$ levels have been found to activate conventional protein kinase C and induce downstream signaling cascades, which in turn affects atrial structural remodeling and promotes the development of AF [25].

## 3. The Molecular and Metabolic Processes Between Ferroptosis and AF

### 3.1 Iron Metabolism

As a form of cell death depending on iron and lipotoxicity, ferroptosis can be triggered by iron overload-related increases in the biogenesis of hydroxyl radicals and other forms of ROS, triggering iron-catalyzed lipid peroxidation. Hydroxyl radicals are generated via the Fenton reaction-mediated reduction of $H_{2}$$O_{2}$ by ferrous iron, and they are particularly deleterious [26]. Yue *et al*. [27] analyzed samples of left ear tissue from patients suffering from persistent AF, and detected significantly higher iron particle levels in these samples via Prussian blue staining consistent with the AF-related dysregulation of iron metabolism. In line with these results, Rose *et al*. [28] observed a selective reduction in CaV1.3-dependent L-type ${\mathrm{Ca}}^{2+}$currents in response to iron overload, potentially raising the risk of AF incidence.

Ferritin heavy chain 1 (FTH1) is a key regulator of the transport and uptake of iron that facilitates iron storage. Reductions in FTH1 expression reportedly contribute to lower levels of stored iron formation such that intracellular ${\mathrm{Fe}}^{2+}$ levels rise, ultimately inducing ferroptosis [29]. Yue *et al*. [27] also noted significant FTH1 downregulation in tissue samples from AF patients, providing additional support for the biological link between ferroptotic death and AF. The same was also noted in a study performed by Dai *et al*. [30], who additionally noted a role for ferroptosis in excessive ethanol intake-induced AF while revealing that the inhibition of such ferroptotic activity was sufficient to partially abrogate AF risk.

Ferroportin (FPN) is another major iron transporter mediating the export of iron ions into the blood, and it is similarly involved in ferroptotic induction. Cardiac FPN is essential for the regulation of iron homeostasis in the cardiac tissue, thus suppressing lipid peroxidation and modulating the incidence of ferroptosis [31]. Fang *et al*. [32] additionally noted that the pathogenesis of lipopolysaccharide (LPS)-induced endotoxemia-related new-onset AF was closely linked to ferroptotic activity. In another recent report, the adverse effects of intracellular iron load were ablated by Shensong Yangxin (SSYX)-mediated FPN upregulation, mitigating the biogenesis of ROS within cells and thereby reversing atrial structural and electrical remodeling to effectively reduce AF susceptibility [33].

In summary, ferroptosis has been shown to affect the development of AF by regulating iron metabolic pathways such as intracellular iron transport proteins and accumulation of ferrous ions, which provides a theoretical basis for the subsequent clinical application of ferroptosis.

### 3.2 Cystine Metabolism

A wealth of evidence now points toward a link between ferroptosis, dysregulated lipid peroxidation, reductions in the activity of GPX4, cystine/glutamate transport system inhibition, and tissue fibrosis [34]. GPX4 is an enzyme that is central to the maintenance of intracellular antioxidant activity, protecting against ferroptotic induction through the conversion of toxic lipid hydroperoxides into less deleterious alcohol and lipid molecules [35]. Specifically, GPX4 functions as a lipase, catalyzing the conversion of GSH to oxidized glutathione (GSSG) through an oxidation reaction. This process is crucial for eliminating excess peroxides and hydroxyl radicals produced during cellular respiration and metabolism, consequently reducing the peroxidation of polyunsaturated fatty acids in cell membranes. Depletion of GSH and diminished GPX4 activity lead to an accumulation of free reactive ferrous iron, which enhances the production of ROS through the Fenton reaction. Increased ROS interact with polyunsaturated fatty acids in lipid membranes, causing lipid peroxidation and ultimately leading to ferroptosis [36].

The solute carrier family 7 member 11 (SLC7A11) and solute carrier family 3 member 2 (SLC3A2) light and heavy chain proteins comprise the ${\mathrm{Xc}}^{-}$ system of cell surface cystine/glutamate reverse transporters, which mediate cystine uptake and the release of glutamate to promote the synthesis of GSH, thereby alleviating oxidative stress and preventing lipid peroxidation-related cell death [37]. Yue *et al*. [27] reported significantly reduced SLC7A11 and GPX4 expression in AF patients, reaffirming the integral role that ferroptotic activity plays in AF-related myocardial fibrosis and suggesting that such activity is mediated by the xCT/GPX4 pathway. Liu *et al*. [38] additionally noted that exosomes derived from fibroblasts during cardiac pacing were capable of promoting ferroptotic induction in atrial fibrillating cardiomyocytes via exo-miR-23a-3p secretion, which is, in turn, able to target SLC7A11 and the ${\mathrm{Xc}}^{-}$ system. This additionally suggests that efforts to inhibit the release of exosomes may help protect against susceptibility to AF [39].

In conclusion, these findings emphasize the influence of amino acid metabolism in ferroptosis-associated AF, which plays an important role in further investigating aspects of the development and progression of AF.

### 3.3 Lipid Oxidation Metabolism

The dysregulation of normal lipid metabolic activity is closely associated with the risk of AF, as it contributes to the excessive biogenesis of oxidative metabolites which causes myocardial dysfunction and related atrial remodeling [40]. Oxidative stress-associated lipid peroxidation is also directly related to the incidence of ferroptotic cell death [41]. Higher ROS levels are reportedly linked to AF incidence through changes in the activity of NOX (nicotinamide dinucleotide phosphate oxidase) both locally within atrial cardiomyocytes and systemically in the serum through mechanisms potentially associated with atrial gap junctions. Specifically, atrial gap junctions can be disrupted by oxidative stress, contributing to aberrant electrical conduction [42]. Members of the connexin (CX) protein family, including CX40 and CX43, play a pivotal role in the establishment of these atrial gap junctions.

In Gemel *et al*. [43] study posited that higher levels of ROS produced by the excessive activation of NOX2 may suppress atrial CX40 and CX43 expression, culminating in atrial remodeling. A study performed in 2019 utilized a lipidomics approach to monitor AF-related shifts in lipid patterns in patient samples, ultimately revealing a link between increased free fatty acid levels and AF incidence [44]. Tham *et al*. [45] further performed a lipidomics-based cohort analysis of 3779 patients in order to better clarify the correlative relationships between particular phospholipids and AF incidence, detecting a particularly close relationship with phosphatidylethanolamine (PE). On the other hand, as an essential component of ferroptosis, Acyl-Coenzyme A synthetase long-chain family member 4 (ACSL4) regulates ferroptosis sensitivity by promoting the conversion of polyunsaturated fatty acids from phospholipids to lipid peroxides [46]. In more recent analyses, Huang *et al*. [47] demonstrated the ability of PE to promote GPX4 downregulation and ACSL4 upregulation, contributing to the induction of ferroptosis in the context of angiotensin II (Ang II)-induced AF.

In brief, ferroptosis induced by abnormal lipid metabolism is closely associated with the development of AF. These mechanisms include overexpression of assembly junction proteins, downregulation of GPX4 expression, upregulation of ACSL4 expression, and increased oxidative metabolites that promote AF.

### 3.4 Abnormal Mitochondrial Damage

Mitochondria are crucial organelles for iron utilization, metabolism, and maintaining intracellular iron homeostasis [6]. Ferroptosis, induced by iron-catalyzed lipid peroxidation, impairs mitochondrial function and exacerbates cardiomyocyte injury. Morphologically, ferroptotic mitochondria exhibit membrane rupture and blistering, reduced or absent mitochondrial cristae, and increased mitochondrial membrane density [5]. Lipid peroxidation compromises cellular membranes, leading to membrane bubbling, a key event in ferroptosis. Additionally, mitochondria are vital for cardiomyocyte function, providing the energy necessary for the heart’s mechanical and electrical activities. Recent research is uncovering the molecular mechanisms by which abnormal mitochondrial damage contributes to cardiomyocyte dysfunction and AF [48]. Furthermore, mitochondria are the primary source of intracellular ROS, which promotes electrical remodeling in AF and induces mitochondrial dysfunction by rapidly diminishing mitochondrial inner membrane potential (ΔΨm) and reducing energy production [49]. Mitochondrial dysfunction is implicated in AF progression, and compounds that protect mitochondrial function have shown efficacy in preventing contractile dysfunction in Drosophila models of AF [50].

In general, cardiomyocyte dysfunction during AF onset may stem from ferroptosis, leading to mitochondrial dysfunction, structural and DNA damage, and electrophysiologic deterioration.

### 3.5 Other Relevant Signaling Pathways

Oxidative stress has a range of effects on cells, resulting in altered intracellular ${\mathrm{Ca}}^{2+}$ homeostasis [51]. Indeed, the biogenesis of high levels of ROS can disrupt normal ${\mathrm{Ca}}^{2+}$ processing such that ${\mathrm{Ca}}^{2+}$ overload occurs [52]. This, in turn, results in the activation of downstream ${\mathrm{Ca}}^{2+}$-dependent pathways and changes in L-type calcium channel subunit expression such that L-type ${\mathrm{Ca}}^{2+}$ currents are suppressed and the duration of action potentials is shortened [53]. When normal ${\mathrm{Ca}}^{2+}$-handling protein dynamics are disrupted, this can lead to consequent changes in intracellular ${\mathrm{Ca}}^{2+}$ transients and diastolic sarcoplasmic reticulum release of ${\mathrm{Ca}}^{2+}$ [54].

The transcription factor nuclear factor-erythroid 2-related factor 2 (NRF2) plays an important role in the regulation of oxidative homeostasis [55], while also being closely associated with ferroptosis [56]. NRF2 plays a pivotal role in mitigating lipid peroxidation, with its target genes, heme oxygenase-1 (HO-1) and quinone oxidoreductase 1 (NQO1), orchestrating oxidative stress response and iron metabolism in cells. The NRF2-HO-1 axis augments ${\mathrm{Xc}}^{-}$ system expression, thereby bolstering cellular resilience against ferroptosis [36]. Significantly, HO-1, the target gene of NRF2, not only possesses antioxidant properties but also elevates ferritin levels, augments the expression and activity of the endoplasmic reticulum’s adenosine triphosphate (ATP)-dependent iron export pumps, facilitates iron efflux, and diminishes intracellular iron concentrations [57]. The administration of exosomes derived from bone marrow mesenchymal stem cells overexpressing NRF2 can activate NRF2/HO-1 pathway to protect against myocardial fibrosis in AF rat model [58]. Fang *et al*. [32] further determined that the NRF2 downstream effector protein FPN is closely associated with ferroptotic induction, exacerbating ${\mathrm{Ca}}^{2+}$ handling-related proteins in the context of LPS-induced endotoxemia and new-onset AF. Yu *et al*. [59] also found that the anti-ferroptotic flavonoid icariin was capable of suppressing SIRT1/NRF2/HO-1 pathway signaling, ultimately suppressing atrial remodeling and AF susceptibility resulting from the consumption of excessively high levels of ethanol.

These findings highlight the involvement of other signaling pathways in the effects of ferroptosis on AF, including changes in intracellular ${\mathrm{Ca}}^{2+}$, the SIRT1/NRF2/HO-1 axis, and the expression of the NRF2 downstream factor FPN. These changes, through their regulatory functions, contribute to the prevention of cardiac oxidative homeostasis, atrial remodeling, and the response of AF to ferroptosis.

## 4. Ferroptotic Inhibitors Offer Potential Value for the Management of AF

In an animal model, Pennell *et al*. [60] found that arrhythmias could develop as a consequence of the iron toxicity-related disruption of normal cardiac electrical condition. The administration of iron chelators to animals suffering from iron overload has further been shown to reduce the incidence of AF and other forms of cardiac arrhythmias [61].

Yu *et al*. [59] noted that the lipid peroxidation inhibitor ferrostatin-1 (Fer-1) was able to attenuate both the generation of mitochondrial ROS and associated atrial remodeling. Icariin, a flavone compound extracted from *Herba epimedii*, was also demonstrated to suppress atrial electrical remodeling induced by ethanol, with concomitant atrial electrical conduction velocity improvements, atrial conduction inhomogeneity reductions, and the attenuation of maladaptive structural remodeling in the atrial compartment, thereby protecting against local tissue injury [59]. Liu *et al*. [38] noted that Fer-1 was able to prevent both oxidative stress-associated damage and ion channel remodeling. These authors additionally noted that GW4869, which is an inhibitor of exosomal biogenesis, was able to prevent the exosome-mediated export of harmful substances from cells while also inhibiting signaling activity downstream of sphingomyelinase to suppress ROS production, thereby lowering rates of cardiomyocyte ferroptosis and delaying electrical remodeling [38]. Yeh *et al*. [62] determined that statin administration was sufficient to reduce AF episodes through the promotion of NRF2/HO-1 upregulation within cardiomyocytes while protecting against oxidative stress tachycardia-induced fibrillation, ultimately suppressing myocardial remodeling.

Liproxstatin-1, an effective ferroptosis inhibitor, has been shown to prevent mitochondrial lipid peroxidation and to restore the expression of GSH, GPX4, and ferroptosis suppressor protein 1. This suggests a theoretical basis for employing ferroptosis inhibitors in the treatment of AF [63]. In addition, natural antioxidants exhibit three key properties: free radical scavenging, iron chelation, and reducing capabilities. Compounds such as curcumin, baicalein, quercetin, puerarin, and phloroglucinols, derived from Hypericum japonicum, have demonstrated ferroptosis inhibition, as highlighted in the review by Shaghaghi *et al*. [64]. This indicates that potent natural antioxidants could serve as potential ferroptosis inhibitors in AF treatment.

On the whole, ferroptosis inhibitors exhibit a range of potential mechanisms for combating AF, such as reducing lipid peroxidation, modulating mitochondrial ROS production, and regulating gene expression, thereby inhibiting atrial remodeling. However, further studies are needed to fully understand and unravel the complexity of these mechanisms.

## 5. Clinical and Future Perspectives

AF is one of the most common cardiac arrhythmias, with high morbidity and mortality rates throughout the globe contributing to an immense clinical burden. The exact mechanisms of AF are still unknown, and it is thus crucial to further define the molecular factors involved in the pathogenesis of AF. Ferroptosis may affect the onset and progression of AF through iron metabolism, amino acid metabolism, lipid metabolism, and other related pathways. The association between ferroptosis and AF is currently in its nascent stage. Presently, most evidence originates from cellular or animal models, with all human-based findings being empirical. Consequently, the possibility that these observations are influenced by factors other than ferroptosis cannot be discounted. Additional cellular, animal model-based, and clinical research examining the deleterious role that ferroptosis plays in AF is thus needed to better establish the relative importance of this form of cell death.

The significance of systemic iron homeostasis abnormalities in AF pathogenesis has garnered increased research interest recently. Ferroptosis, an emerging form of non-apoptotic cell death, involves iron-dependent lipid peroxidation. Interestingly, both iron overload in ferroptosis and iron deficiency can precipitate AF development. Anemia, commonly associated with various cardiac disorders, predominantly results from iron deficiency. A study indicates a higher anemia prevalence in patients with permanent AF than in those with paroxysmal or persistent AF [65]. Consequently, dynamically assessing iron metabolism balance is vital for understanding AF progression. Meanwhile, iron homeostasis at the cellular level is maintained through the coordinated post-transcriptional regulation of proteins related to iron uptake, export, and storage, including ferritin [66]. Abnormal deposition of ferritin results in an excessive accumulation of iron, making the measurement of serum ferritin levels valuable for evaluating the risk and prognosis of patients with AF. In general, future research focused on cardiomyocyte iron metabolism may offer a novel approach to AF prevention.

## 6. Conclusions

**Fig. 1.**
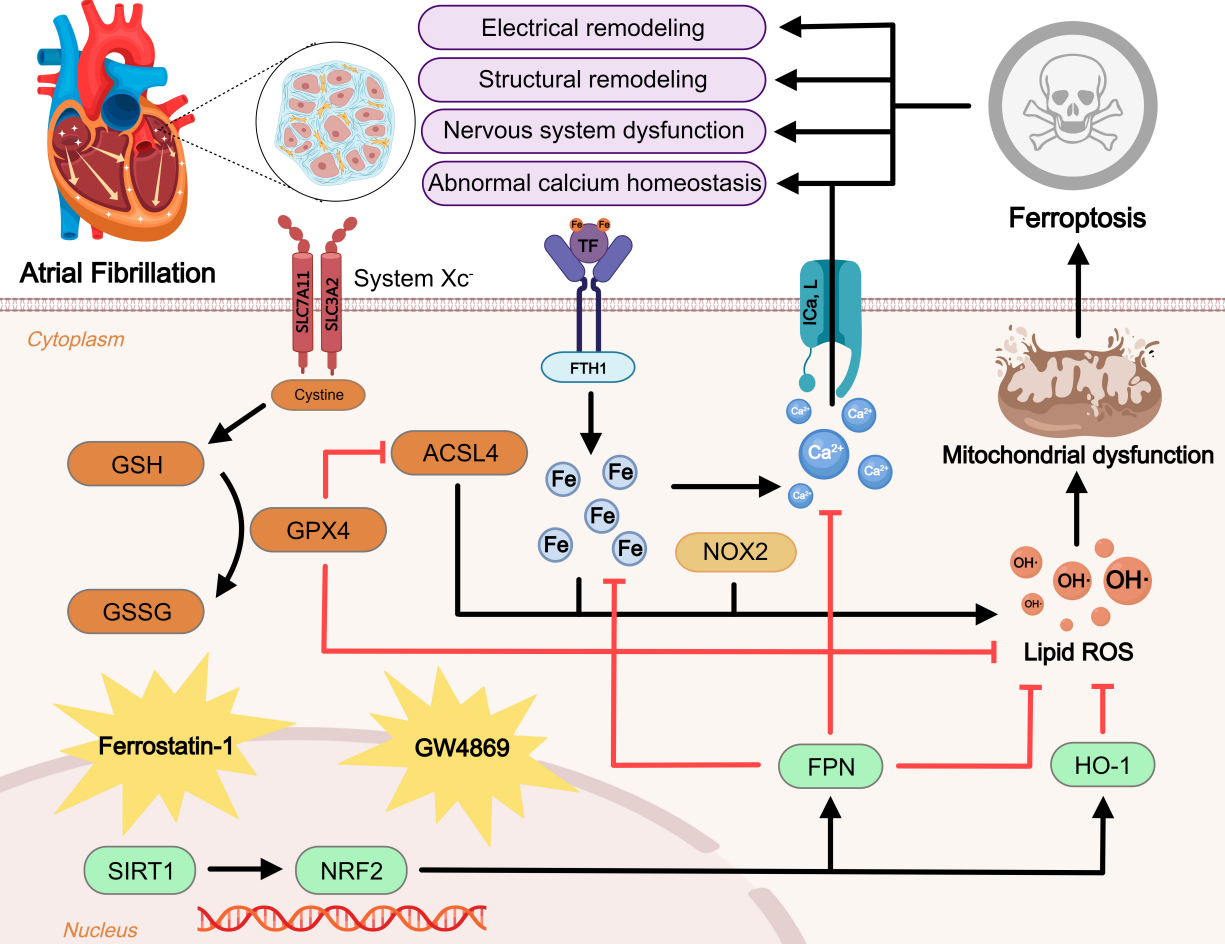
**Regulatory mechanism of ferroptosis in atrial fibrillation (AF). **Ferroptosis is a novel form of iron-dependent cell death. Excessive iron uptake or reduced iron excretion leads to intracellular iron overload, which promotes the Fenton reaction and intracellular peroxide accumulation. GPX4 is an important regulator of the Xc-GSH-GPX4 axis system that blocks ferroptosis. In addition, ROS generated by ACSL4 and NOX2 overactivation promote lipid peroxidation. These three metabolic pathways lead to mitochondrial dysfunction and promote ferroptosis, further exacerbating AF. NRF2, an antioxidant factor, is regulated by SIRT1 and can inhibit ferroptosis by manipulating its downstream factors. At the same time, ferroptosis leads to disturbances in intracellular calcium homeostasis and promotes the onset and progression of AF. Inhibitors of ferroptosis including ferrostatin-1 and GW4869 are displayed. Blunt-ended lines indicate inhibition while arrows indicate promotion. GSH, glutathione; GPX4, glutathione peroxidase 4; GSSG, glutathione disulfide; ACSL4, acyl-CoA synthetase 4; SLC7A11, solute carrier family 7 member 11; SLC3A2, solute carrier family 3 member 2; TF, transferrin; FTH1, ferritin heavy chain 1; NOX2, reduced nicotinamide adenine dinucleotide phosphate oxidase 2; $I_{\text{Ca,L}}$, L-type ${\mathrm{Ca}}^{2+}$ current; OH⋅, reactive free radicals; ROS, reactive oxygen species; SIRT1, sirtuin 1; NRF2, nuclear factor erythroid-2-related factor 2; FPN, ferroportin; HO-1, heme oxygenase 1.

Overall, this review provides an overview of the current understanding of how ferroptosis contributes to the pathogenesis of AF while also discussing the potential value of ferroptotic inhibitors as an approach to treating this condition (as Fig. 1). The research completed to date highlights the promise of developing targeted inhibitors of cardiomyocyte ferroptosis as a means of preventing AF, providing a strong foundation for these ongoing analytical efforts.
